# Explicating the Cognitive Process of a Physician’s Trust in Patients: A Moderated Mediation Model

**DOI:** 10.3390/ijerph192114446

**Published:** 2022-11-04

**Authors:** Qijun He, Yungeng Li, Zhiyao Wu, Jingjing Su

**Affiliations:** 1 School of Journalism and Communication, Shanghai University, Shanghai 200072, China; 2 School of Media and Communication, Shanghai Jiao Tong University, Shanghai 200240, China; 3 School of Nursing, The Hong Kong Polytechnic University, Kowloon, Hong Kong

**Keywords:** physician’s trust in patient, perceived integrity of patient, perceived ability of patient, communication efficacy, moderated mediation model

## Abstract

Trust is considered a critical factor in the physician–patient relationship. However, little is known about the development and impact of physicians trusting their patients. A model that is premised on the integrated model of organizational trust was proposed in this article to reveal the cognitive processes involved in physicians’ trust, with perceived integrity and the ability of the patient as antecedents and the physicians’ communication efficacy as the outcome. A cross-sectional survey of 348 physicians in Zhejiang province, China, revealed that a physician’s trust in a patient mediated the relationship between the physicians’ perception of the integrity and ability of the patient, and the physician’s communication efficacy. The physicians’ educational backgrounds and work experience were also found to moderate an indirect effect: a lower level of education and longer work experience intensified the impact of the perceived integrity and ability of the patient on the physician’s trust, while shorter work experience made the association between the physician’s trust and communication efficacy more salient. This paper provided implications for both physician and patient sides.

## 1. Introduction

Trust plays a vital role in the physician–patient relationship in terms of influencing healthcare delivery [1]. It is defined as “an expectation that the other person will behave in a way that is beneficial, or at least not harmful” [2] (p. 148). The focus of previous studies has been mainly on the patients’ perspective while examining the determinants and impact of patients’ trust in physicians [3,4]. The studies on physicians’ trust are scarce probably because the patients are usually considered the more vulnerable party and the physicians are in dominant position to exert their power and authority [5,6]. Therefore, the patients’ perspectives are enhanced by and in turn result in an emphasis on professionalism in healthcare [7]; physicians are obligated to trust the patients as a professional requirement and medical ethics, and physicians’ trust is treated as a state of “ought to be” rather than as an actual fact [6]. Besides, the patients’ trust in physicians has direct influence on the medical outcome through patients’ cooperation and involvement, adherence to medical recommendations, satisfaction with medical care, etc. [8]; accordingly, the direct practical implications can be drawn from the patients’ perspective trust studies.

However, it is important to investigate the physicians’ trust [6]. It has been found to be common that, in reality, physicians do not trust their patients [9,10]. Physicians’ trust in their patients cannot simply be increased and maintained through morality or training, but should be considered a socio-psychological concept which is associated with the physicians’ vulnerability [11]. The physicians’ vulnerability arises from uncertainty: the uncertainty about the patient’s cooperation and the medical outcome [12] and, more importantly, the uncertainty from the risk that physicians face various types of workplace violence. According to ILO/ICN/WHO/PSI Joint Programme, 8% to 38% of healthcare professionals globally have experienced physical violence being visited on them by patients and visitors; the ratio of suffering threat or verbal aggression is even higher [13]. Therefore, the high level of physicians’ trust is not naturally guaranteed, especially when the physicians’ dominance is weakened in the increasingly shared decision-making and coordinated healthcare processes [6]. Moreover, physicians’ trust in patients is critical to the therapeutic process and medical outcome. Physicians’ distrust affects physicians’ psychological state and service quality [14,15]; by contrast, physicians who have higher levels of trust in patients might disclose more information, provide a better medical service, and enhance their patients’ trust and cooperation [5,11]. To understand the influencing factors and the outcome of physicians’ trust is therefore both necessary and important, especially when the cooperation and interaction facet of physician–patient relationship has been increasingly emphasized in the past two decades [4,16].

In the existing literature of physicians’ trust in patients, more attention is paid to the impact of physicians’ (dis)trust on medical and relationship outcomes [3,6,11,12], while few studies focus on the nuanced cognitive mechanism at the individual level, that is, the development and the consequential influence of physicians’ trust in patients. This cognitive process of physicians’ trust is worth closer examination because it examines the physicians as ordinary people who are affected by various factors of the environment and also make decisions based on their cognitive judgment. Without analysis of the cognitive development and consequences of physicians’ trust, a piece of the puzzle is, therefore, missing in physician–patient relationship studies. During the COVID-19 pandemic, the risk of virus exposure and the heavy workload have increased the healthcare professionals’ stress level, which may further affect their trust and professional performance [17,18]. Therefore, the aim of this study was to examine the cognitive process of physicians’ trust from a socio-psychological perspective.

Chinese physicians were sampled as the research object because the cultural and societal context of China puts physicians in a dilemma of trust; particularly in terms of culture, the diagnosis and treatment in traditional Chinese medicine rely on the mutual trust and the close relationship between physicians and patients, and trust is therefore stressed very much in traditional Chinese medical ethics [19]. The traditional cultural traits such as guanxi (personal ties) also enhance the role of trust in physician–patient relationships [20]. On the other hand, the expansion of the healthcare system in China in the past decade has increased the possibility of physician–patient conflict and distrust [21,22]. A meta-analysis revealed that approximately 62.4% of the healthcare professionals in China have experienced various types of workplace violence [23]. The majority of the perpetrators were patients and their families, and the common causes were the poor outcomes of the medical service (e.g., death, sequelae, and misdiagnosis), miscommunication, and the medical charges [24,25]. This study, thus, provides contextual insight variance into the non-Western experience by examining the cognitive process of physicians’ trust in a relatively perplexing context [12].

### 1.1. Theoretical Background and Hypothesis Development

#### 1.1.1. The Integrated Model of Organizational Trust

Although shaped by interactions, the development and consequences of trust are largely a cognitive process: the trustor develops trust based on the trust belief generated by specific situations and the trustees; the trust, then, determines one’s behavioral intention toward the trustee [26,27]. The integrated model of organizational trust [28] has been widely adopted to explore the cognitive process of trust in the workplace [29]. Taking a trustor’s perspective, the model presents the perceived trustworthiness of the trustee—consisting of ability, benevolence, and integrity—as the antecedent of trust, while the behavior-related cognitive consequences of being a trustor, such as willingness to take a risk in the relationship, represents the outcomes. Moreover, in terms of this model, the personal traits of the trustor and the contextual factors should also be considered.

Just as the physician’s credibility and behavior can affect the patient’s trust [30,31,32,33,34], so can the patient’s characteristics impact the physician’s trust [35]. The perceived integrity and ability of the patient are considered to influence the patient’s perceived trustworthiness [9]. The perceived integrity of the patient refers to the patient’s intention to comply with principles and regulations, and to be unlikely demonstrate excessive or unethical behaviors in a medical context. This perception is influenced by the patient’s socioeconomic status, deviant behavior, moral and legal consciousness, and psychological state, among other factors [36,37,38,39,40]. The physician doubting the integrity of a patient is the most salient factor of physicians’ distrust in China [10]. Second, the perceived ability of the patient indicates the patient’s capability to correctly communicate and process medical information during the encounter, as well as to follow the physician’s instructions. This perception is influenced by factors such as the patient’s educational background, medical literacy, prior medical knowledge, and self-management ability [39,41,42,43,44]. According to the integrated model, physicians develop a perception of the trustworthiness of the patient on the basis of the abovementioned two antecedents. The perceived benevolence of the trustee, defined as “a trustee is believed to want to do good to the trustor, aside from an egocentric profit motive” [28] (p. 718), is not suitable in the physician–patient relationship because the physicians are not supposed to benefit directly from the patients. As such, the benevolence factor in the original model is not discussed here. Therefore, H1 was proposed as:

**H1**: *The physician’s perceived integrity (H1.1) and the perceived ability (H1.2) of the patient are positively associated with a physician’s trust in a patient.*

As for outcome, the physician’s communication efficacy was analyzed as the behavior-related cognitive consequence of trust. Communication efficacy refers to one’s belief that one can successfully perform communication behaviors with others, which is determined by the perceived consequences of certain communication behaviors [45]. A perceived negative outcome would trigger a self-protection intention and result in lower communication efficacy, while trust in others could enhance one’s communication efficacy because it could mitigate negative expectations and self-guarding [46].

Self-efficacy is a key concept in social cognitive theory because it links predictive knowledge with decision-making and behaviors [47]. It could be the abovementioned missing piece of the puzzle. That is, it could explain the possible cognitive cause of objective medical and relational outcomes: The physician’s (dis)trust does not directly result in certain medical or relational outcomes, but rather does so indirectly through the physicians’ communication efficacy and consequential communication. While the authors of the existing literature mainly believe that physicians’ effective communication relies on communication skills, and therefore stress the cultivation of skills, the skills are performed better when physicians have higher trust and confidence levels in themselves with respect to successfully performing communication behaviors; that is, they have higher communication efficacy [48,49]. The physician’s trust in the patient determines their enacted trust and is closely associated with their self-trust [11]. In this vein, a physician’s trust in the patient is expected to influence their communication efficacy. H2, H3, and the research question were, therefore, proposed as follows:

**H2**: *The physician’s trust in the patient is positively associated with the physician’s communication efficacy.*

**H3**: *The physician’s trust in the patient mediates the relationship between physician’s perceived integrity (H3.1), and the perceived ability (H3.2) of the patient and the physician’s communication efficacy.*

**RQ**: *Does the physician’s perception of the integrity and ability of the patient directly influence their communication efficacy?*

#### 1.1.2. The Moderating Role of the Physician’s Education and Work Experience

In relationships, trust varies across people [29,50]. The integrated model of organizational trust encouraged scholars to explore the moderating effect of the trustor’s personal characteristics in order to not only examine the robustness of the model, but also explicate the nuanced individual differences in trust process [28]. In previous studies, individual factors, such as trust propensity, age, gender, and uncertainty, were found to have moderating effects on the path from trustworthiness to trust or from trust to behavioral intentions [51,52,53,54].

Physicians have reported differing levels of confidence in building trust with their patients, and the individual characteristics are worth exploring [55]. Education and work experience, as two frequently mentioned factors in physician–patient relationship studies, are particularly considered as the moderators of the mediation paths here, because physicians’ attitude, trust and communication toward patients, as professional ethics or skills, are associated with training and practice [56,57]. In this vein, compared with other characteristics such as gender and level of hospital [10,58], education, and work experience could help to examine the influence of professional training and practice to the physicians’ implicit trust process. On the one hand, physicians with higher education levels might tend to have lower trust in patients but higher communication efficacy level [14,58,59], despite some studies suggesting the opposite [10]. On the other hand, physicians who have longer work experience might have higher trust in both the patient and in themselves, as well as better communication skills [10,11,60,61], but some studies showed physicians’ longer work experience was associated with more biased attitudes, less trust, or less efficacy toward certain patients [62,63]. There is scarce evidence, however, of the effect of these two factors on the whole process, from perception of the patient to communication through trust. Therefore, it is worth exploring a potential moderating effect of physician’s education and work experience. H4 and H5 were proposed as follows:

**H4**: *Education moderates the indirect effect of a physician’s perception of the integrity (H4.1) and ability (H4.2) of the patient on the physician’s communication efficacy through the physician’s trust in the patient.*

**H5**: *Work experience moderates the indirect effect of a physician’s perception of the integrity (H5.1) and ability (H5.2) of the patient on the physician’s communication efficacy through physician trust in the patient.*

**Figure 1:**
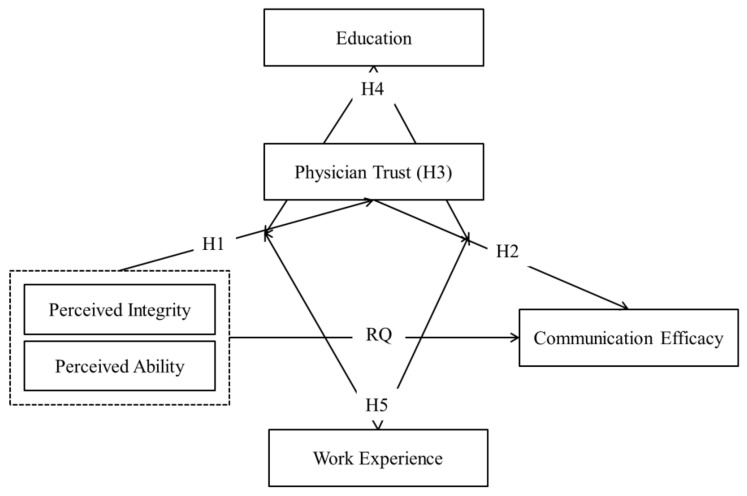
The conceptual model.

The conceptual model of the study is shown in Figure 1.

## 2. Materials and Methods

### 2.1. Participants

Data from a cross-sectional survey were utilized to evaluate the conceptual model. Physicians from four hospitals in Zhejiang Province were selected to participate in the study, which took place from November 2021 to March 2022. Zhejiang province was chosen because it represented the abovementioned two characteristics of Chinese healthcare reform. As one of the most developed provinces in China, Zhejiang has high levels of both medical quality and patient-initiated workplace violence [64]. The sample included physicians from various departments, including outpatients, orthopedics, gynecology, and obstetrics. The selection criteria were that the doctors had to be regular physicians who were officially contracted to the selected hospitals, doing clinical work, and voluntarily participated in the survey. The participants were asked to complete the questionnaire individually after informed consent had been obtained. A total of 450 questionnaires were distributed and 436 were returned. Thus, the response rate was 96.9%. However, only 348 out of 436 physicians completed their questionnaires, which were retained for the following analysis. The participants’ demographics are shown in Table 1.

### 2.2. Measures

#### 2.2.1. Perceived Integrity of the Patient

The items were developed from previous studies [28,65]. The respondents were asked to recall the last patients they had encountered and to indicate their agreement on four items: “the patient obeyed the medical rules and kept order,” “the patient did not seek privilege through guanxi (personal ties) or personal positions,” “the patient did not have a tendency or behavior to be violent towards medical staff,” and “the patient did not only care about the interests of themselves.” The items were rated on a 5-point Likert scale, ranging from 1 (not at all) to 5 (very much) (*M* = 3.25, *SD* = 1.13, Cronbach’s α = 0.906).

#### 2.2.2. Perceived Ability of the Patient

These items were also developed from previous studies [28,65]. The respondents were asked to indicate their level of agreement with four items: “the patient had basic medical knowledge”, “the patient did not have unrealistic expectations of the medical outcome”, “the patient had a certain understanding and awareness of the disease”, “the patient did not use the experience from unreliable sources (such as Baidu, friends, or folk knowledge) to question your diagnosis.” The items were rated on a 5-point Likert scale, ranging from 1 (not at all) to 5 (very much) (*M* = 2.86, *SD* = 1.19, Cronbach’s α = 0.936).

#### 2.2.3. Physician’s Trust in the Patient

Thom [2]’s Physician Trust in the Patient Scale was utilized to measure the physicians’ trust. The respondents were asked to indicate their confidence in their last patients on 12 items, such as: “Provide all the medical information you need?” and “Respect your time?” The items were rated on a 5-point Likert scale, ranging from 1 (not confident at all) to 5 (very confident) (*M* = 4.16, *SD* = 0.47, Cronbach’s α = 0.962).

#### 2.2.4. Communication Efficacy

The items were adapted from Zhao et al.’s and Wei’s studies [66,67]. The respondents were asked to indicate their agreement with five items, such as “I have a full understanding of the patient’s condition after communicating with the patient” and “I can explain clearly the pros and cons of various treatment options to the patient”. The items were rated on a 5-point Likert scale, ranging from 1 (not at all) to 5 (very much) (*M* = 3.42, *SD* = 1.00, Cronbach’s α = 0.783).

#### 2.2.5. Moderator

The participants’ educational backgrounds (1 = junior college or below; 2 = bachelor’s degree; 3 = master’s degree; 4 = doctoral degree) and work experience as a physician (1 = within 5 years; 2 = 6–10 years; 3 = 11–20 years; 4 = above 20 years) were measured as moderators.

#### 2.2.6. Controls

The participants’ ages, genders, professional titles, and general perceptions of their physician–patient relationships (ranging from 1 = very bad to 5 = very good) were included as control variables.

## 3. Results

SPSS PROCESS version 3.3 (SPSS Inc., Chicago, IL, USA) was utilized to test the research hypotheses and to answer the research question [68]. The mediation effect, namely H1, H2, H3, and RQ, was tested by Model 4 in PROCESS, and the moderated mediation effect, namely H4 and H5, was tested by Model 58 in PROCESS. The effects were tested with 5000 bootstrap samples at 95% bias-corrected confidence intervals [69].

### 3.1. Mediation Effect

Two mediation effect models were conducted: The perceived integrity and perceived ability of the patient were entered as the independent variables, while trust in the patient served as the mediator and communication efficacy as the outcome variable. Age, gender, professional title, and general perception of the physician–patient relationship were entered as covariates.

For the perceived integrity of the patient, the total effect model was significant (*R*^2^ = 8.29%, *F* = 6.18, *p* < 0.001). Perceived integrity was positively related to physician trust (*B* = 0.33, *SE* = 0.04, *p* < 0.001), and physician trust was positively related to communication efficacy (*B* = 0.11, *SE* = 0.04, *p* < 0.05). The indirect effect of perceived integrity on communication efficacy through trust was significant (effect = 0.04, *SE* = 0.02, CI [0.01, 0.07]), whereas the direct effect was not significant (effect = −0.01, *SE* = 0.03, CI [−0.07, 0.06]), indicating a full mediation effect. Among the control variables, age (*B* = 0.08, *SE* = 0.04, *p* < 0.05) and professional title (*B* = 0.08, *SE* = 0.04, *p* < 0.05) showed positive significance in predicting communication efficacy.

For the perceived ability of the patient, the total effect was also significant (*R*^2^ = 8.66%, *F* = 6.48, *p* < 0.001). Perceived ability was positively associated with physician trust (*B* = 0.36, *SE* = 0.04, *p* < 0.001), and physician trust was positively associated with communication efficacy (*B* = 0.10, *SE* = 0.04, *p* < 0.01). The indirect effect of perceived ability on communication efficacy through trust was also significant (effect = 0.04, *SE* = 0.02, CI [0.01, 0.07]), and the direct effect was not significant (effect = 0.01, *SE* = 0.03, CI [−0.05, 0.07]), suggesting a full mediation effect. Among the control variables, professional title (*B* = 0.07, *SE* = 0.04, *p* < 0.05) was positively related to communication efficacy, and age showed marginal significance (*B* = 0.07, *SE* = 0.04, *p* = 0.08).

Therefore, H1, H2, and H3 were supported. To answer the RQ, no direct effect was found for the physician’s perception of the integrity and ability of the patient on communication efficacy.

### 3.2. Moderated Mediation Effect

The moderated mediation effect indicated that the mediation process was dependent on a moderator variable [68]. To test H4 and H5, four moderated mediation models were performed. In the first two models, education was used as the moderator, while the perceived integrity and perceived ability of the patient were entered as the independent variables; trust in the patient served as the mediator, and communication efficacy as the outcome variable. In the second two models, work experience was the moderator, and the others remained the same. For each model, age, gender, professional title, and general perception of the physician–patient relationship were entered as covariates.

As shown in Table 2, in the test related to H4, the interaction effect of education and perceived integrity on physician trust was significant (*B* = −0.14, *SE* = 0.04, *p* < 0.001), while the interaction effect of education and trust on communication efficacy was not significant (*B* = 0.04, *SE* = 0.03, *p* > 0.05). The association between perceived integrity and physician trust was stronger for physicians with low levels of education (simple slope = 0.43, *t* = 8.87, *p* < 0.001) than for those with high levels of education (simple slope = 0.22, *t* = 4.31, *p* < 0.001). As shown in Table 2, the conditional, indirect effect of perceived integrity on communication efficacy through physician trust was significant when the level of education was high (effect = 0.03, *SE* = 0.01, CI [0.01, 0.06]), but insignificant when the level of education was low (effect = 0.04, *SE* = 0.02, CI [−0.01, 0.08]).

Second, the interaction effect of education and perceived ability on physician trust was marginally significant (*B* = −0.07, *SE* = 0.05, *p* = 0.08); the association between perceived ability and physician trust was stronger for physicians with low levels of education (simple slope = 0.41, *t* = 9.14, *p* < 0.001) than for those with high levels of education (simple slope = 0.31, *t* = 6.03, *p* < 0.001). As shown in Table 2, the conditional, indirect effect of perceived integrity on communication efficacy through physician trust was significant when the level of education was high (effect = 0.04, *SE* = 0.02, CI [0.01, 0.08]), but insignificant when the level of education was low (effect = 0.03, *SE* = 0.02, CI [−0.01, 0.07]). The moderated mediation effect of education was confirmed, and H4 was supported.

Table 3 shows the results for H5. Similarly, the interaction effect of work experience and perceived integrity on physician trust was significant (*B* = 0.25, *SE* = 0.05, *p* < 0.001). The association between perceived integrity and physician trust was stronger for people with longer work experience (simple slope = 0.52, *t* = 9.54, *p* < 0.001) than for those with shorter work experience (simple slope = 0.17, *t* = 3.27, *p* < 0.01). The interaction effect of work experience and physician trust on communication efficacy was also significant (*B* = −0.12, *SE* = 0.04, *p* < 0.01), but the association between physician trust and communication efficacy was stronger for people with shorter work experience (simple slope = 0.20, *t* = 4.19, *p* < 0.001) than for those with longer work experience (simple slope = 0.04, *t* = 0.80, *p* > 0.05). As shown in Table 3, the conditional, indirect effect of perceived integrity on communication efficacy through physician trust was significant when work experience was short (effect = 0.03, *SE* = 0.02, CI [0.01, 0.07]), but insignificant when work experience was long (effect = 0.02, *SE* = 0.03, CI [−0.04, 0.07]).

Meanwhile, the interaction effect of work experience and perceived ability on physician trust was significant (*B* = 0.10, *SE* = 0.05, *p* < 0.05); the association between perceived ability and physician trust was stronger for people with longer work experience (simple slope = 0.42, *t* = 8.49, *p* < 0.001) than for those with shorter work experience (simple slope = 0.27, *t* = 4.60, *p* < 0.001). As shown in Table 3, the conditional, indirect effect of perceived ability on communication efficacy through physician trust was significant when work experience was short (effect = 0.05, *SE* = 0.02, CI [0.02, 0.09]), but insignificant when work experience was long (effect = 0.01, *SE* = 0.02, CI [−0.03, 0.05]). Thus, the moderated mediation effect of work experience was confirmed and H5 was supported.

## 4. Discussion

The authors drew on the integrated model of organizational trust to examine the trust relationship between physicians and patients. The study contributed to the physician–patient relationship field by revealing the process of cognitive trust development from the physician’s perspective, as well as by providing empirical understandings in a non-Western context.

First, a cognitive path was built from the perceived integrity and ability of the patient to the physician’s trust in the patient, and then to the physicians’ communication efficacy. The physician’s trust in the patient served as the mediator between the antecedents and outcome. The mediation effect remained significant when moderators were included. This study was conducted as a response to the call for micro-level investigations of physician’s trust [11] and added a missing link between physician–patient interaction and medical outcome. That is, physician’s trust is not natural or stable, or acquired by emphasizing medical professionalism. Rather, as human beings, the building of the physicians’ trust is a dynamic process that develops during physician–patient communication, and has an impact on the communication process. Meanwhile, the findings reflected the contextual influence because the physicians’ perceptions of patients were not based merely on occasional individual encounters, but on past experience or prior relationships with other patients [12].

More specifically, the findings also provide a comparison of the influence of the abovementioned two origins of physician trust—the uncertainty of medical outcome and the risk of workplace violence [2,12,14,15]. Compared with the perceived integrity of the patient, the perceived ability of the patient had slightly larger explanatory power regarding physician’s trust. This suggests that, when building trust in patients, the physicians are concerned more about the uncertainty of the medical outcome than the risk of workplace violence. Given that the mean of perceived ability was significantly lower than perceived integrity, the finding suggests the influence of the patients’ health literacy on medical outcomes from the physician’s perspective. Previous studies were primarily examinations of the medical consequences of low health literacy from the patient’s perspective, such as communication difficulties, poor patient adherence, and high hospitalization rates [70,71,72]. This study, however, indicates the possible path from health literacy to medical outcomes via the cognitive process of the physicians and buttresses the importance of enhancing health literacy in China [73,74].

The influence of the physician’s individual characteristics on the trust process was also examined. The moderating role of a physician’s education background and work experience on the trust process was confirmed. The results showed that lower education and longer work experience intensified the impact of the perceived trustworthiness of the patient on the physician’s trust, whereas shorter work experience made the association more salient between physician trust and communication efficacy. The fluctuation of physicians’ trust level was less associated with their perceptions toward the patients when they were with higher level of education. The possible explanation is that professional training could help reduce implicit bias during medical encounter [56], which indicated the necessity of physicians’ professional training [7]. However, the relatively small effect size indicated other possibilities should be considered in further studies; for example, physicians with higher levels of education were probably more confident about the medical outcome, and accordingly their trust level was not influenced by their perceptions much. The findings also highlighted the importance of work experience. Work experience might exercise a stabilizing influence on physicians’ communication efficacy when their trust level fluctuated; that is, the experienced physicians’ self-confidence over communication performance is less dependent on their trust in patients. The possible explanation is, although communication skills can be learned, there is a gap between training and the authentic experience [75]. Therefore, physicians with longer work experience could practice physician–patient communication more, which helped them manage to perform communication skills at most occasions. Moreover, age and professional title also contributed to the increase of communication efficacy.

## 5. Conclusions

Based on the integrated model of organizational trust, this study examined the cognitive process of physicians’ trust in patients with antecedents, outcome, and moderating factors. The Chinese sample adopted in this study adds knowledge to the realm with the non-Western contextual variance [12]. As mentioned above, the physicians in China face a dilemma that trust is culturally emphasized but practically limited. Accordingly, the physicians may be cautious when developing and performing trust: they prudentially decide which patients to trust, and feel more intimate and confident with the trustworthy patients. Therefore, trust plays a more critical role in the physicians’ cognitive path in Chinese context compared with that in other social contexts; in this vein, the Chinese sample may represent a salient scenario of how the physicians’ trust works.

The results have practical implications for patients, physicians, and institutions. For patients, as the physicians build trust based on their perceptions of their patients’ ability and integrity, it is suggested that in order gain a decent health service, the patients should (1) enhance their health literacy in order to improve their comprehension when seeking medical advice and (2) cultivate their understanding of physicians’ professionalism and standpoint so that they can behave themselves when seeking professional help. On the physician and institution side, it is suggested that the physicians should (1) acknowledge the importance of trust process and adjust the possible bias or mistrust in their daily practice and (2) practice to manage communication skills in order to build stable interactions with patients. On the institution side, it is suggested that (1) communication skills and conflict management training should be provided during both medical education and practice and (2) the rights and safety of physicians should be protected, which will establish a more physician- and patient-friendly medical service environment.

There are several limitations of this study. First, although significant, the effect sizes of the models were relatively small; additional antecedents such as the institutional and systematic factors should be included in future studies to achieve enhanced explanatory power. Second, more moderators could be included to explicate the individual variance, such as the physician’s propensity to trust, perceived risk of trust, etc. Third, the generalizability of the findings might be undermined by the representative limitation of the sample. Future researchers should investigate the development of physician’s trust by incorporating more factors with a more-representative sample.

## Figures and Tables

**Table 1:** Demographics.

Variable	Frequency	Percentage %
*Gender*		
Man	178	51.1
Woman	170	48.9
*Age*		
18–25	57	16.4
26–35	125	35.9
36–45	145	41.7
Above 45	21	6.0
*Professional title*		
Intern	105	30.2
Resident physician	124	35.6
Physician in charge	105	30.2
Associate professor or professor	14	4.0
*Education*		
Junior college or below	64	18.4
Bachelor’s degree	175	50.3
Master’s degree	97	27.9
Doctor’s degree	12	3.4
*Work experience*		
Within 5 years	158	45.4
6–10 years	146	42.0
11–20 years	38	10.9
Above 20 years	6	1.7

**Table 2:** Conditional indirect effect of perceived integrity and perceived ability of the patient on communication efficacy through physician trust moderated by education.

IV	Mediator	Condition	Conditional Indirect Effect of Education		
			Effect	*SE*	95% CI
Perceived integrity of patient	Physician trust	Low (−1 SD)	0.0352	0.0210	[−0.0050 to 0.0786]
		Middle (0)	0.0372	0.0149	[0.0103 to 0.0692]
		High (+1 SD)	0.0326	0.0138	[0.0088 to 0.0621]
Perceived ability of patient	Physician trust	Low (−1 SD)	0.0326	0.0203	[−0.0056 to 0.0748]
		Middle (0)	0.0404	0.0161	[0.0097 to 0.0735]
		High (+1 SD)	0.0449	0.0165	[0.0123 to 0.0777]

Note: Bootstrap resample = 5000. Conditions for the moderator (physician trust) are the mean and plus/minus one standard deviation from the mean. IV = independent variable; *SE* = standard error; CI = confidence interval.

**Table 3:** Conditional indirect effect of perceived integrity and perceived ability of the patient on communication efficacy through physician trust moderated by work experience.

IV	Mediator	Condition	Conditional Indirect Effect of Work Experience		
			Effect	*SE*	95% CI
Perceived integrity of patient	Physician trust	Low (−1 SD)	0.0332	0.0153	[0.0078 to 0.0668]
		Middle (0)	0.0410	0.0155	[0.0125 to 0.0730]
		High (+1 SD)	0.0191	0.0270	[−0.0362 to 0.0693]
Perceived ability of patient	Physician trust	Low (−1 SD)	0.0520	0.0169	[0.0207 to 0.0869]
		Middle (0)	0.0390	0.0143	[0.0101 to 0.0675]
		High (+1 SD)	0.0125	0.0218	[−0.0348 to 0.0528]

Note: Bootstrap resample = 5000. Conditions for the moderator (physician trust) are the mean and plus/minus one standard deviation from the mean. IV = independent variable; *SE* = standard error; CI = confidence interval.

## Data Availability

Not applicable.
